# Acute IL-1RA treatment suppresses the peripheral and central inflammatory response to spinal cord injury

**DOI:** 10.1186/s12974-020-02050-6

**Published:** 2021-01-06

**Authors:** Abi G. Yates, Trisha Jogia, Ellen R. Gillespie, Yvonne Couch, Marc J. Ruitenberg, Daniel C. Anthony

**Affiliations:** 1 Department of Pharmacology, The University of Oxford, Mansfield Road, Oxford, UK; 2 School of Biomedical Sciences, Faculty of Medicine, The University of Queensland, St Lucia, Queensland Australia; 3 Acute Stroke Programme, RDM-Investigative Medicine, The University of Oxford, Oxford, UK; 4 Sechenov First Moscow State Medical University, Moscow, Russia

**Keywords:** Neurotrauma, Systemic inflammatory response syndrome, Acute phase response, IL-1 receptor antagonist, Neutrophils, Macrophage

## Abstract

**Background:**

The acute phase response (APR) to CNS insults contributes to the overall magnitude and nature of the systemic inflammatory response. Aspects of this response are thought to drive secondary inflammatory pathology at the lesion site, and suppression of the APR can therefore afford some neuroprotection. In this study, we examined the APR in a mouse model of traumatic spinal cord injury (SCI), along with its relationship to neutrophil recruitment during the immediate aftermath of the insult. We specifically investigated the effect of IL-1 receptor antagonist (IL-1RA) administration on the APR and leukocyte recruitment to the injured spinal cord.

**Methods:**

Adult female C57BL/6 mice underwent either a 70kD contusive SCI, or sham surgery, and tissue was collected at 2, 6, 12, and 24 hours post-operation. For IL-1RA experiments, SCI mice received two intraperitoneal injections of human IL-1RA (100mg/kg), or saline as control, immediately following, and 5 hours after impact, and animals were sacrificed 6 hours later. Blood, spleen, liver and spinal cord were collected to study markers of central and peripheral inflammation by flow cytometry, immunohistochemistry and qPCR. Results were analysed by two-way ANOVA or student’s t-test, as appropriate.

**Results:**

SCI induced a robust APR, hallmarked by elevated hepatic expression of pro-inflammatory marker genes and a significantly increased neutrophil presence in the blood, liver and spleen of these animals, as early as 2 hours after injury. This peripheral response preceded significant neutrophil infiltration of the spinal cord, which peaked 24 hours post-SCI. Although expression of IL-1RA was also induced in the liver following SCI, its response was delayed compared to IL-1β. Exogenous administration of IL-1RA during this putative therapeutic window was able to suppress the hepatic APR, as evidenced by a reduction in CXCL1 and SAA-2 expression as well as a significant decrease in neutrophil infiltration in both the liver and the injured spinal cord itself.

**Conclusions:**

Our data indicate that peripheral administration of IL-1RA can attenuate the APR which in turn reduces immune cell infiltration at the spinal cord lesion site. We propose IL-1RA treatment as a viable therapeutic strategy to minimise the harmful effects of SCI-induced inflammation.

## Introduction

Systemic inflammatory response syndrome (SIRS) is a major and frequent complication in trauma patients with a spinal cord injury (SCI). Development of SIRS not only has the potential to cause multi-organ damage and/or dysfunction [1, 2], it is also thought to influence inflammatory pathology and lesion site development in the injured central nervous system (CNS) itself, including following brain or spinal cord insult. Previous studies from our lab have demonstrated that the latter is heavily influenced by the magnitude of early systemic inflammatory changes, a process referred to as the acute phase response (APR), which predominantly occurs in the liver [3–7]. Here, pro-inflammatory cytokines and chemokines as well as acute phase proteins (APPs) are upregulated as early as 2 hours post-injury [5, 7–10]. These mediators contribute to the mobilisation and priming of leukocytes, facilitating their translocation to sites of CNS injury.

Neutrophils are the first peripheral immune cells to be recruited to CNS lesion sites, including in SCI [11], and they are known to release proteases and generate oxidative species [12, 13]. In peripheral trauma, this enables degradation of debris which is beneficial to the host. However, in the CNS, this causes further membrane damage, lipid peroxidation, DNA fragmentation and cellular injury [14]. Activated neutrophils also release pro-inflammatory mediators, which can contribute to the development of a chronic immune response [15, 16] and exacerbate the neurological deficit [17].

Interleukin-1β (IL-1β) is a key early component of the APR and cytokine cascade initiated after SCI. Undetectable in the uninjured cord [18, 19], this pro-inflammatory cytokine is significantly upregulated within 1 hour of injury [20–23], and thought to play a pivotal role in the recruitment of various leukocyte subsets [24–26]. Early inhibition of IL-1β signalling could therefore be of significant benefit. IL-1 receptor antagonist (IL-1RA) is an endogenous competitive antagonist that inhibits IL-1 signalling and subsequent downstream pro-inflammatory events [18]. IL1-RA expression in the injured CNS is initially downregulated compared to IL-1β [27], but its exogenous administration can lead to improvements in cognitive deficits [28, 29], tissue loss [29] and leukocyte recruitment [30, 31] in models of traumatic brain injury (TBI) and stroke. IL-1RA has also been used in models of SCI [32–34] but the focus, as with studies in the brain, has tended to be on its suppressive effects in the CNS only. With recent papers demonstrating important interactions between migrating neutrophils and components of the CNS immune system [35], a better understanding of the peripheral effects of IL-1RA treatment is highly significant as it potentially negates the need to circumvent the precarious blood-CNS barrier.

In this paper we investigated the peripheral immune response to a lower thoracic SCI and determined how treatment with the endogenous antagonist IL-1RA impacted on the APR and neutrophil recruitment to the injured spinal cord.

## Methods

### Animals

Female C57BL/6J mice, 8-12 weeks old, were used in this study, as described previously [36, 37], due to reduced risk of post-operative complications. Animals were obtained from the Animal Resource Centre (Canningvale, Western Australia) and housed in individually ventilated cages in a specific pathogen-free facility, under standard diurnal lighting conditions (12 hours) with *ad libitum* access to food and water. The experimental holding room had temperature (21.5 °C set point) and humidity control (40% set point), and was supplied with HEPA-filtered air. Animals were allowed to acclimatise for 4 weeks prior to experiments. All experiments were approved by The University of Queensland’s Animal Ethics Committee (Anatomical Biosciences) and were conducted in accordance with the Australian Code for the Care and Use of Animals for Scientific Purposes.

### Spinal cord injury model

Mice were anaesthetised by intraperitoneal injection using a combination of tiletamine/zolezepam (50mg/kg; Virbac) and xylazine (10mg/kg; Troy Laboratories). Following incision of the skin over the lower thoracic region of the back, the paravertebral muscles were separated and anatomical landmarks utilised to identify T9 [38]. A dorsal laminectomy was then performed to expose the spinal cord [39–41], after which the vertebral column was clamped for stabilisation and a moderate contusive 70kDyne (kD) SCI inflicted using the Infinite Horizon Impactor (Precision Systems and Instrumentation). Muscle and skin were then closed with 6-90 polygalactin dissolvable sutures (Ethicon) and Michel wound clips (Kent Scientific), respectively. Age- and weight-matched sham-operated controls were subjected to laminectomy only. All mice received a single dose of buprenorphine in Hartmann’s sodium lactate (1mg/kg; sub cutaneous; Sigma Aldrich) post-surgery. Naïve control mice, which were included for baseline results, did receive anaesthesia but no surgical intervention.

### IL-1RA treatment

Animals received two intraperitoneal injections of human IL-1RA (100mg/kg), the first immediately following impact whilst still under general anaesthesia, and the second 5 hours post-injury. This treatment regime was based on previous papers reporting neuroprotective effects of IL-1RA in models of CNS injury [30, 42].

### Sample collection and processing

For time-course analysis, animals were allowed to survive for 2, 6, 12 and 24 hours post-surgery. For IL-1RA experiments, animals were culled 6 hours post-surgery.

All mice were anaesthetised with 4% isoflurane and blood was collected by cardiac puncture into a heparinised tube (BD Microtainer). Mice were then transcardially perfused with 20mL of heparinised saline (0.9% NaCl, 10IU/mL heparin (Pfizer), 2% NaNO_3_) and fresh liver and spleen samples collected for qPCR. Liver was snap-frozen in liquid nitrogen and stored at -80°C, whilst spleen was processed immediately for flow cytometry (see below). Mouse cadavers were then transcardially perfused with 30mL of Zamboni’s fixative (2% picric acid, 2% paraformaldehyde, pH 7.2-7.4), and fixed liver and spinal column samples removed and stored in fixative overnight at 4°C. Spinal cords were extracted from the columns the following day and post-fixed at 4°C for an additional 24 hours. Tissues were cryoprotected (overnight incubations in 10% and 30% sucrose solution), embedded in optimal cutting temperature compound (OCT, ProSciTech), then snap-frozen in dry ice-cooled 2-methyl-butane and stored at -80°C until further use. Fixed liver (10μm) and spinal cord (transverse, 20μm) were sectioned using a Leica Cryostat (CM1850).

### Flow cytometry

Fresh spleen samples were mechanically dissociated through a 70μm cell strainer (Life Technologies) and splenocytes pelleted through low-speed centrifugation (300*g* for 10 minutes). Cells were then resuspended in 5mL of red blood cell (RBC) lysis buffer, incubated for 5 minutes at room temperature (RT), before being centrifuged again and resuspended in Dulbecco’s PBS (DPBS); 5 million splenocytes per sample were used for analysis. Blood samples were diluted 1:5 in RBC lysis buffer for 5 minutes at RT, after which they were centrifuged and resuspended in 100μl of DPBS for analysis.

To exclude dead cells, splenocyte and blood samples were incubated with Zombie Green or Zombie Near Infrared (1:100 in DPBS), respectively, for 20 minutes at RT in the dark. CD16/32 F_C_ block (BD Biosciences), diluted 1:200 in blocking buffer (0.5% Bovine serum albumin [BSA], 2mM EDTA in DPBS), was used to prevent non-specific antibody binding. Samples were immunolabelled for 20 minutes in the dark with Alexa Fluor® 647 rat α-mouse Ly6G (1A8; 1:100; BioLegend), V450 rat α-mouse Ly6C (AL-21; 1:100; BD Horizon), PE rat α-mouse F4/80 (1:50; BD Biosciences) and BV 785^TM^ rat α-mouse/human CD11b (M1/70; 1:100; BioLegend) for spleen, or PE rat α-mouse/human CD11b (M1/70; 1:200; BioLegend) for blood. Next, 1mL of blocking buffer was added and the cells isolated by low-speed centrifugation at 4°C. Cell pellets were resuspended in blocking buffer and then analysed using the LSR II flow cytometer (BD Biosciences) with BD FACS Diva software.

Clumped cells, debris and dead cells were removed during the gating process; neutrophils and monocytes were defined as the CD11b^+^SSC^hi^Ly6G^+^ and CD11b^+^SSC^lo^F4/80^lo^Ly6G^-^ populations, respectively. Propidium iodide-fluorescing counting beads (10μl; Beckman Coulter) were added to each sample to enable the absolute quantification of cell numbers.

### Immunostaining and quantification

For Immunofluorescent staining, fixed spinal cord sections were blocked for non-specific binding with 10% BSA in PBS with 0.3% Triton X-100 for 1 hour at RT. Sections were then incubated overnight at 4°C with primary antibody (rat α-Ly6B.2, 1:200, Bio-Rad). The following day, sections were washed in PBS and incubated in secondary antibody (donkey α-rat 594, 1:400, Jackson Immunoresearch) and Hoechst 33342 nuclear dye (1:1000, Sigma) for 1 hour at RT. Sections were then washed again, mounted with DAKO fluorescent mounting medium (Sigma) and coverslip.

For immunohistochemical staining with the chromogenic reporter 3,3’-Diaminobenzidine (DAB), fixed liver and spinal cord sections were first blocked for endogenous peroxidase activity (1% H_2_O_2_ in methanol) and biotin (1:20 in PBS, Vector Labs), followed by incubation with 10% goat serum in PBS for 1 hour at RT to reduce non-specific protein binding. Sections were then incubated overnight at 4°C with primary antibody (rabbit α-MBS neutrophils, 1:10,000, made in house; rabbit α-IBA-1, 1:2000, Abcam), diluted in 1% goat serum in PBS. Next, sections were incubated in goat α-rabbit biotinylated secondary antibody (1:200; Vector Labs) for 2 hours at RT, followed by ABC (1:100; ThermoFisher) for 1 hour at room temperature. After several rounds of washing, sections were incubated in DAB until a satisfactory level of staining was achieved; 1% Harris Haemotoxylin or cresyl violet was used as a counterstain for liver and spinal cord, respectively. Stained sections were dehydrated through graded alcohols (80%, 90%, 2x100%, 5 minutes each), cleared with xylene (2x5 minutes), mounted with non-aqueous mounting medium and coverslipped.

For quantification, stained cells in liver sections were counted directly using a light microscope (Leitz Dialux 20) with an eyepiece grid at x40 magnification, across three sections, in three representative fields per section. For the spinal cord, immunopositive neutrophils were counted at the lesion epicentre and for ±1.0mm in rostral and caudal direction using the either Nikon Eclipse upright automated stereology and slide scanning microscope with Stereo Investigator software (immunofluorescent staining), or the Leitz Dialux 20 light microscope (immunohistochemical staining). All cell counting was performed blinded.

### RNA extraction and qPCR

RNA was extracted from approximately 30mg of snap-frozen fresh liver (Qiagen© RNEasy Mini Kit). RNA concentration was measured using a NanoDrop (ThermoFisher) and 1000ng of sample was converted to cDNA using the Applied Biosystems High Capacity cDNA conversion kit. Real-time qPCR was performed with duplicates for each sample (10-25ng/well) using SYBR green qPCR master mix (Applied Biosystems or PrimerDesign) and the Applied Biosystems QuantStudio Flex 7 or Roche LightCycler 480. The following primers were purchased from PrimerDesign: GAPDH (F: AACGACCCCTTCATTGAC, R: TCCACGACATACTCAGCAC), RPL13a (F: GAGGTCGGGTGGAAGTACCA, R: TGCATCTTGGCCTTTTCCTT), IL-1β (F: CAACCAACAAGTGATATTCTCCAT, R: GGGTGTGCCGTCTTTCATTA), CXCL1 (F: GCTGGGATTCACCTCAAGAAC, R: TGTGGCTATGACTTCGGTTTG), CXCL10 (F: CATCCCGAGCCAACCTTCC, R: CACTCAGACCCAGCAGGAT), SAA-2 (F: TGGCTGGAAAGATGGAGACAA, R: AAAGCTCTCTCTTGCATCACTG) and IL-1Rn (F: GACCTTTTACCTGAGAAACAAC, R: TAGGCACCATGTCTATCTTTTC). Relative expression was determined by the 2^-ΔΔCt^ method and normalisation to housekeeping genes (GAPDH and RPL13a).

### Statistical analysis

All analysis was completed with GraphPad Prism 7 software. Unpaired student’s t-test, one- and two-way analysis of variance (ANOVA) were employed, with post-hoc testing as appropriate. Results were considered significant at p<0.05 with 95% confidence intervals. All quantitative data are expressed as mean ± standard error of the mean (SEM).

## Results

### Surgery induces a robust but differential APR in the liver of sham and SCI mice

**Fig 1.**
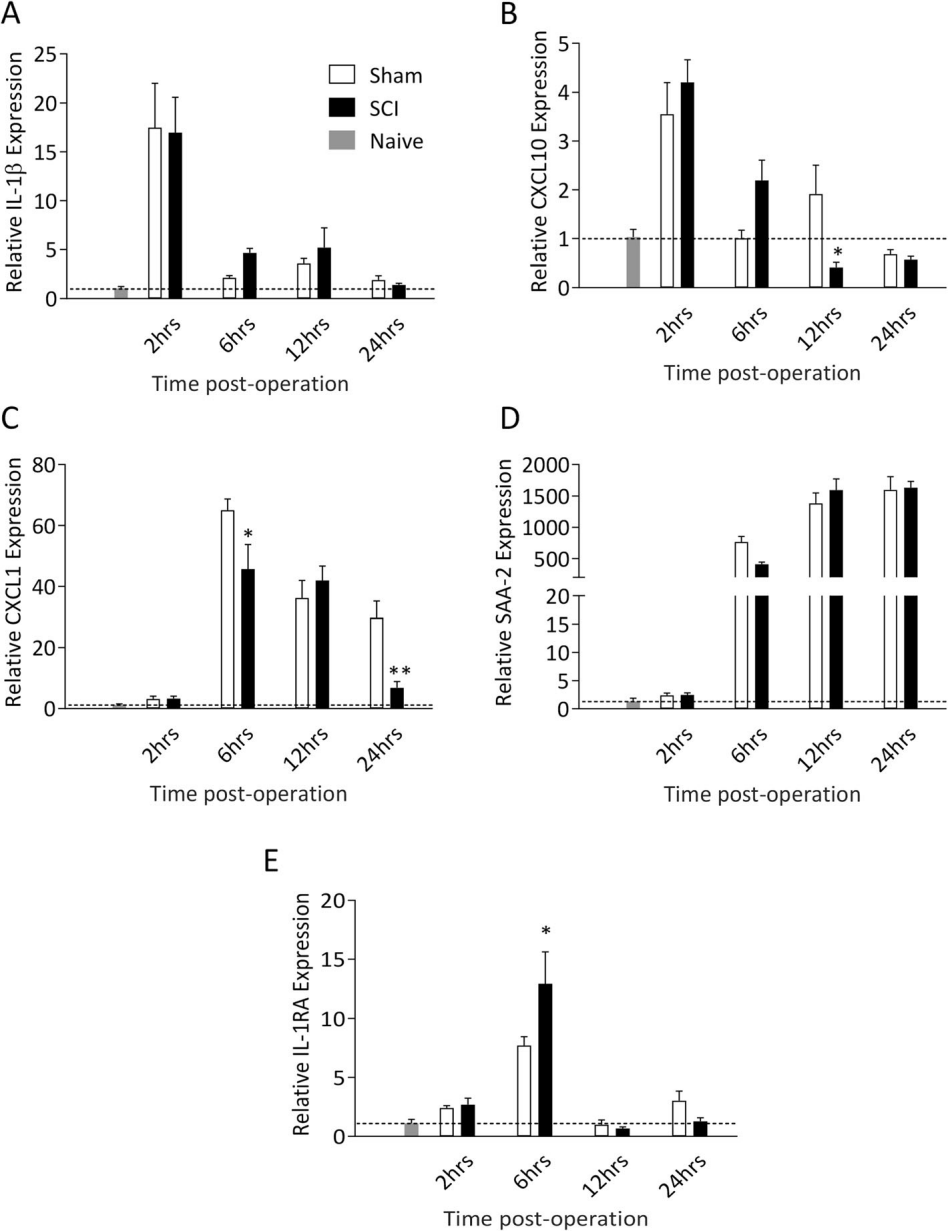
Spinal cord injury induces expression of inflammatory genes in the liver. Female C57BL/6 mice underwent either a 70kD SCI (black bars, *N*=4-5/time point) or sham surgery (white bars, *n*=5/time point). Tissue was collected at 2, 6, 12 and 24 hours post-operation and compared to naïve baseline (grey bar and dotted line, *n*=5). Relative hepatic expression levels of inflammatory genes IL-1β (**a**), CXCL10 (**b**), CXCL1 (**c**) and SAA-2 (**d**), and the IL-1 receptor antagonist IL-1RA (E) were determined by qPCR. Data are presented as mean ±SEM and were analysed by two-way ANOVA. *p<0.05, **p<0.01 with post-hoc test

We first evaluated the temporal profile of the APR in response to a moderate T9 SCI. Previous work from our group has demonstrated that the level of expression of the mRNA for acute phase proteins is a reliable measure of the acute phase response by the liver, and that the levels reflect the subsequent protein expression levels [5, 9]. The relative expression of IL-1β mRNA (Fig. 1a) and CXCL10 mRNA (Fig. 1b) was elevated in both the sham and SCI groups at 2 hours post-injury. By 6 hours, the expression of these mRNA species had returned to naïve baseline levels in the sham controls, but they remained increased in the SCI mice (IL-1β p<0.01, CXCL10 p<0.05; student’s t-test). Expression of CXCL1 was increased in all operated mice (Fig. 1c), yet it was significantly decreased in the SCI mice compared to the sham controls at both 6 (p<0.05) and 24 hours (p<0.01) post-injury. Expression of SAA-2 (Fig. 1d) was also significantly increased over naïve baseline levels in both sham and SCI mice, although there was a trending decrease in the SCI animals at 6 hours post-injury; direct comparison at this time point indeed revealed a significant difference (p<0.01), which was lost when correcting for multiple comparisons (*p*=0.182). Lastly, hepatic expression of IL-1RA, the endogenous antagonist to IL-1, was also significantly increased in response to SCI (6 hours post-injury, p<0.05; Fig. 1e), however, peak expression was delayed compared to that of IL-1β expression. Thus, there was a selective increase in the injury-induced expression of specific APR elements (i.e. IL-1β and CXCL10) following SCI while others (CXCL1 and SAA-2) appeared to be suppressed compared to the sham-operated controls. Taken together with the IL-1RA results, these findings highlight the pro- and anti-inflammatory aspects of the SIRS response.

### The APR of SCI mice is associated with an increased presence of myeloid cells in the liver that precedes the timing of their recruitment to the spinal cord

**Fig 2.**
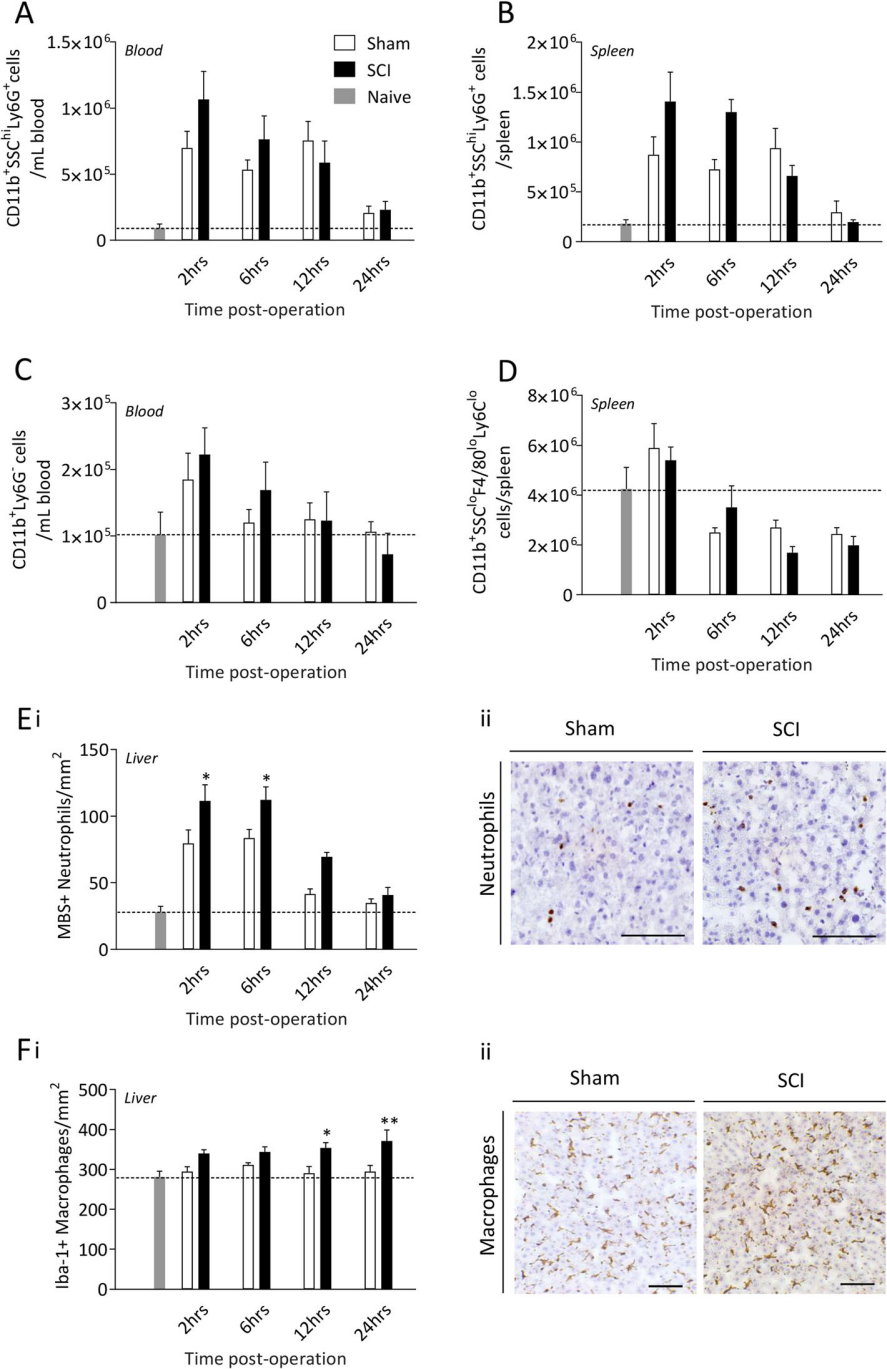
Spinal cord injury induces leukocyte mobilisation in the periphery. Female C57BL/6 mice underwent either a 70kD SCI (black bars, *n*=4-5/time point) or sham surgery (white bars, *n*=5/time point). Tissue was collected at 2, 6, 12 and 24 hours post-operation and was compared to naïve baseline (grey bar and dotted line, *n*=5). Neutrophil mobilisation in the blood (**a**) and spleen (**b**), and monocyte mobilisation in the blood (**c**) and spleen (**d**) was determined by flow cytometry. Neutrophil (**e**) and macrophage (**f**) density in the liver was measured by immunohistochemistry. Representative images of neutrophil and macrophage density in each injury group at peak immune cell density; neutrophil and macrophage density were greatest at 2 and 24 hours post-SCI respectively. Scale bar represents 50μm. Data are presented as mean ±SEM and were analysed by two-way ANOVA, *p<0.05, **p<0.01 with post-hoc test

**Fig 3.**
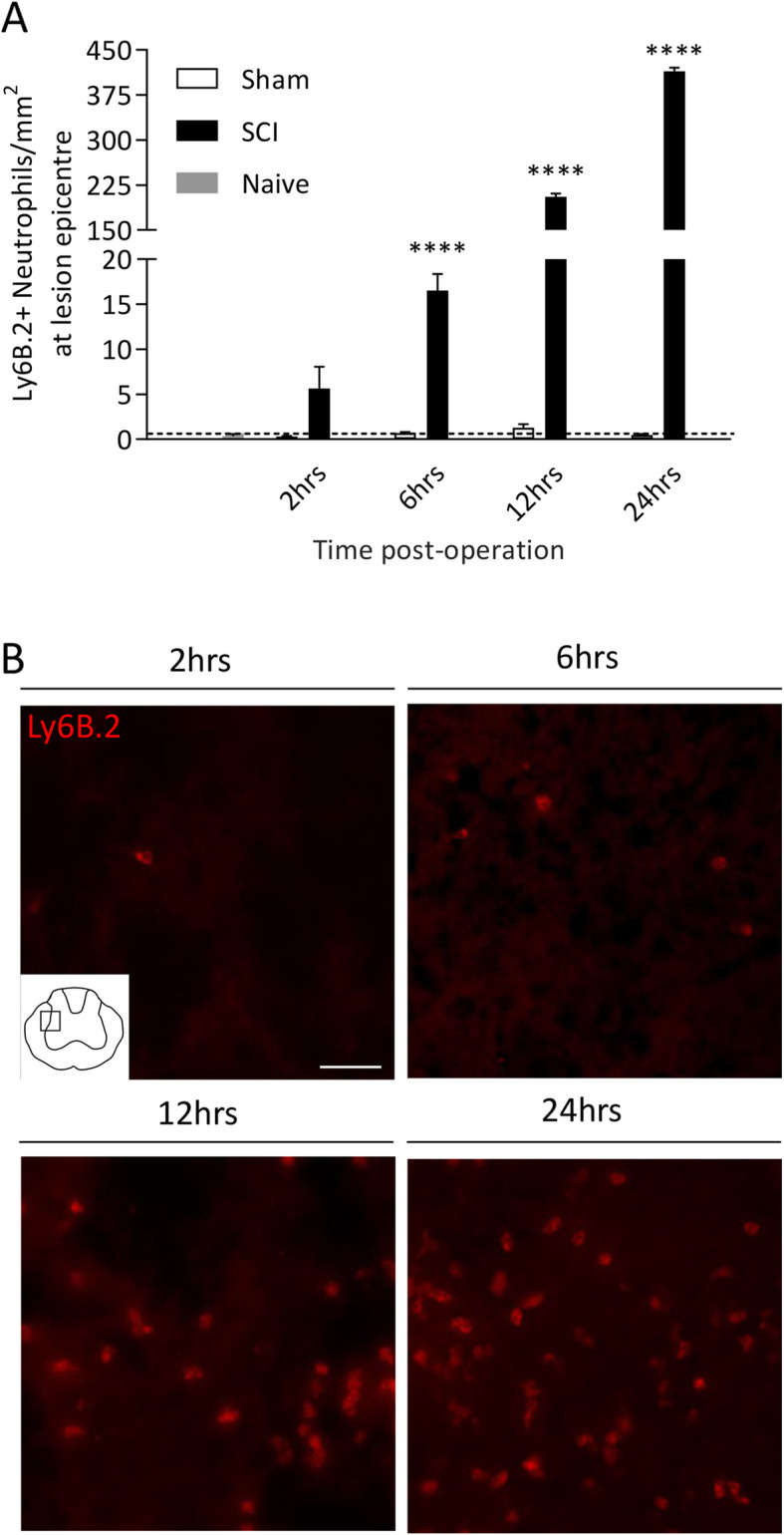
**Neutrophil infiltration of the spinal cord lesion peaks 24 hours post-injury.** Female C57BL/6 mice underwent either a 70kD SCI (black bars, *n*=4-5/time point) or sham surgery (white bars, *n*=5/time point). Tissue was collected at 2, 6, 12 and 24 hours post-operation and was compared to naïve baseline (grey bar and dotted line, *n*=5). Neutrophil cell density was calculated at the lesion epicentre (**a**). Representative images of neutrophil density in the epicentre of spinal cord lesions at each time point (**b**). Scale bar represents 50μm. Data are presented as mean ±SEM. Two-way ANOVA, ****p<0.0001 with post hoc test

We next employed both flow cytometry and immunohistochemistry to investigate downstream myeloid cell responses in the blood, spleen and liver. Circulating CD11b^+^SSC^hi^Ly6G^+^ granulocytes, representing predominantly neutrophils and a small proportion of basophils and eosinophils, were significantly elevated over naïve baseline in both sham and SCI mice at 2, 6, and 12 hours post-surgery (Fig. 2a). CD11b^+^SSC^hi^Ly6G^+^ granulocytes were also increased acutely (2-12 hours) in the spleens of all operated mice (Fig. 2b), with trending increases in SCI mice at both 2 (*p*=0.104) and 6 (p=0.072) hours post-surgery. CD11b^+^Ly6G^-^ monocytes in the blood were increased in all animals above naïve at 2 hours post-surgery (Fig. 2c). At 6 hours, circulating monocytes in sham animals returned to baseline, but they remained elevated in SCI animals, although this was not statistically significant. In the spleen, the number of CD11b^+^SSC^lo^F4/80^lo^Ly6C^lo^ monocytes showed a non-significant increase at 2 hours, and they then decreased at 6-24 hours in all animals compared to naïve controls (Fig. 2d). Acute increases in (MBS^+^) neutrophil numbers were also observed in the livers of both sham and SCI mice, although a greater cell density was observed in animals with neurological injury (p<0.05 at 2 and 6 hours, and *p*=0.055 at 12 hours; Fig. 2e), before returning to baseline control levels by 24 hours post-surgery. The number of Iba-1^+^ monocytes/macrophages was also elevated in the livers of SCI, but not of sham mice, and they progressively increased over the first 24 hours post-surgery (p<0.05 at 12 hours, p<0.01 at 24 hours; Fig. 2f). Finally, while the liver neutrophil response to SCI (or sham surgery) did mostly subside by 24 hours post-surgery, recruitment/presence of these cells at the spinal cord lesion site continued to increase dramatically over the same time period (p<0.0001; Fig. 3).

### Acute treatment with exogenous IL-1RA attenuates the liver APR and reduces neutrophil recruitment to the injured spinal cord

**Fig 4.**
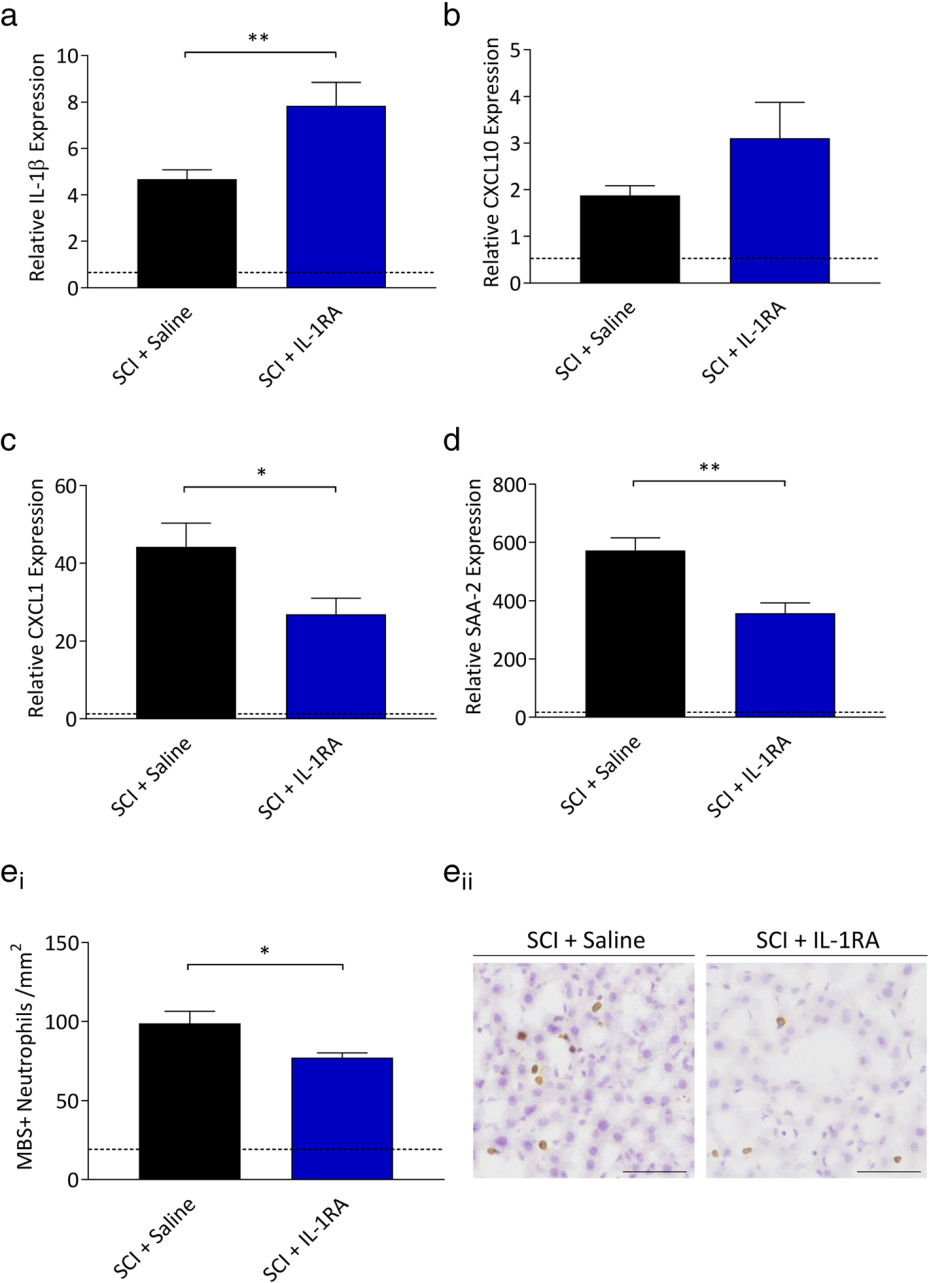
IL-1RA treatment significantly attenuates peripheral inflammation 6 hours post-injury. Female C57BL/6 mice underwent a 70kD SCI and were treated with human IL-1RA (i.p. 100mg/kg, *n*=8) immediately following, and 5 hours after impact, or saline as control (*n*=9). Tissue was collected 6 hours post-injury and compared to naïve baseline (dotted line, *n*=5). Relative expression of IL-1β (**a**), CXCL10 (**b**), CXCL1 (**c**) and SAA-2 (**d**) in the liver, were measured by qPCR. Neutrophil infiltration of the liver was determined by immunohistochemistry (**e**_i_). Representative images of neutrophils staining in the liver (**e**_ii_). Scale bar represents 50μm. Data are presented as mean ±SEM and were analysed by student’s unpaired t-test, *p<0.05, **p<0.01

**Fig 5.**
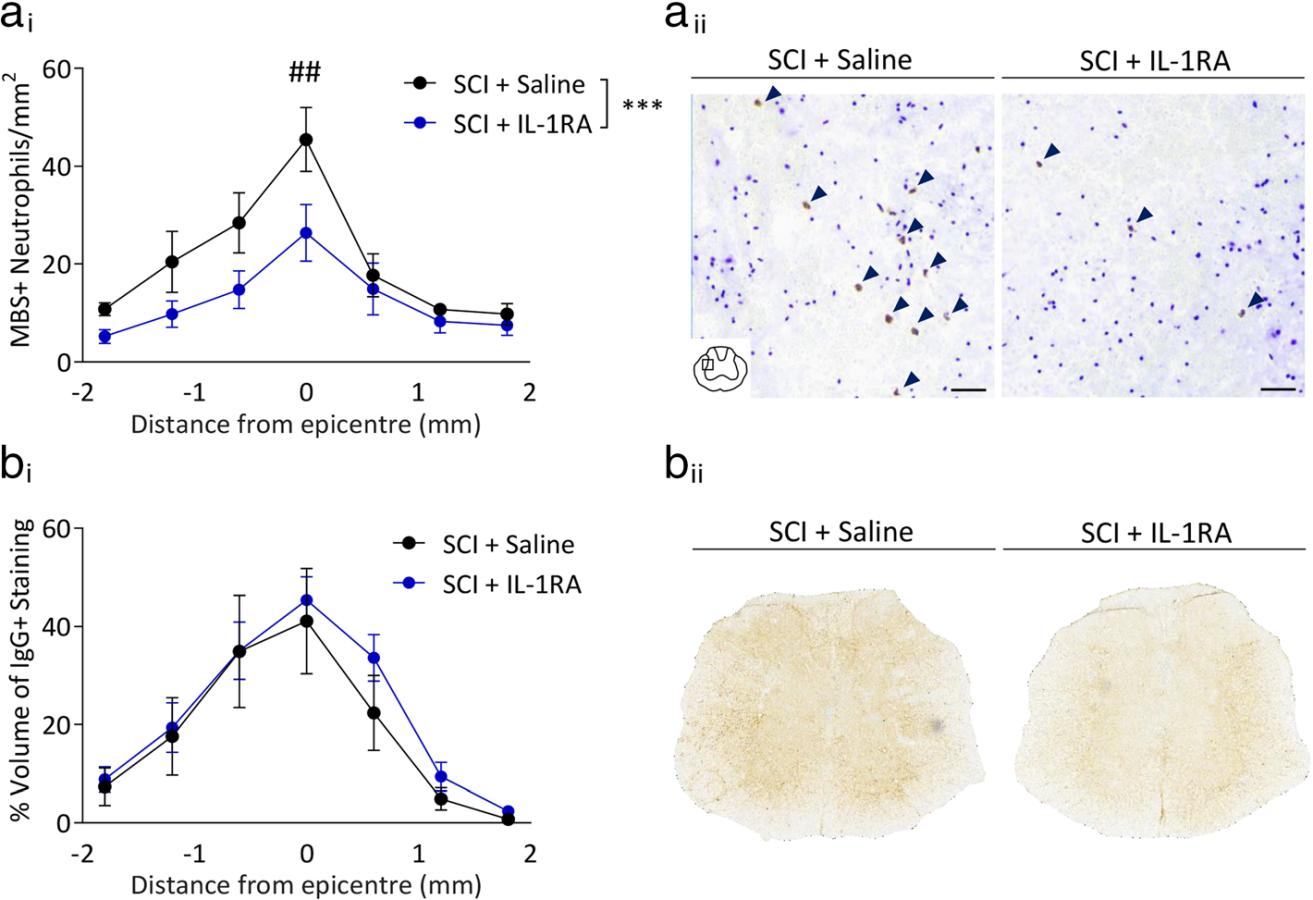
IL-1RA treatment reduces neutrophil recruitment to the injured spinal cord, but not IgG infiltration, 6 hours post-injury. Female C57BL/6 mice underwent a 70kD SCI and were treated with human IL-1RA (i.p. 100mg/kg, n=8) immediately following, and 5 hours after impact, or saline as control (*n*=4). Tissue was collected 6 hours post-injury and fixed spinal cord was stained for infiltrating neutrophils and IgG. Neutrophil cell density was calculated at the epicentre and 3 sections either side, corresponding to ±1.0mm (**a**_i_). Representative images of neutrophil density in the epicentre of spinal cord lesions with, and without IL-1RA treatment, scale bar represents 50μm (**a**_ii_). Volume of infiltrating IgG was measured as a percent of volume of spinal cord (**b**_i_). Representative images of IgG staining 0.6mm from the lesion epicentre (**b**_ii_). Data are presented as mean ±SEM. ***p<0.0001 in two-way ANOVA, ##*p*<0.01 with post-hoc test

Given the time delay between IL-1β and the reciprocal IL-1RA expression, we investigated the effect of supplementing IL-1RA on inhibiting the IL-1β-mediated peripheral inflammatory response. For this, human IL-1RA (100mg/kg) was administered intraperitoneally immediately after contusive SCI and then again 5 hours later. IL-1RA treatment induced an increase in hepatic IL-1β expression (p<0.01; Fig. 4a) while CXCL10 expression was unchanged (*p*=0.126; Fig. 4b). However, significant decreases in the expression of CXCL1 (p<0.05; Fig. 4c) and SAA-2 (p<0.01; Fig. 4d) were observed. Consistent with this attenuated APR, the infiltration of neutrophils into the liver was also significantly decreased in IL-1RA-treated SCI mice (p<0.05; Fig. 4e). We lastly evaluated the effect of IL-1RA treatment on central inflammation, i.e. at the spinal cord lesion site itself (Fig. 5). Strikingly, the SCI-induced recruitment of neutrophils here was significantly attenuated by IL-1RA treatment (p<0.001; Fig. 5a). Intraparenchymal IgG staining was not different between groups (Fig. 5b), demonstrating that injury severity was comparable between groups and also that neutrophil recruitment to the lesion site is an active process.

## Discussion

We have consistently demonstrated that insults to the CNS induce a systemic response, known as the APR, that both precedes and amplifies local inflammation at the lesion site [5, 9]. In this study, we investigated the initiation and progression of the APR during the acute phase of SCI (≤24 hours), and whether this could be therapeutically targeted with peripherally administered IL-1RA. We show, to our knowledge for the first time, that systemic IL-1RA treatment indeed suppresses peripheral inflammation in response to SCI, and that this is in turn associated with reduced neutrophil recruitment to the lesion site.

Our previous work showed that CNS insults lead to the rapid induction of pro-inflammatory cytokines, chemokines and acute phase proteins in the liver as early as 2 hours post-injury [5, 7–10]. These immune mediators are thought to trigger and/or augment the mobilisation and priming of leukocytes in reservoirs such as the bone marrow [1, 36], after which they translocate to both peripheral organs and the site of CNS injury [43]. The present study demonstrates that a robust APR also occurs in mice with a lower thoracic (T9) contusive SCI, with acute increases in hepatic expression of a range of pro-inflammatory genes and an increase in the circulating leukocyte count, particularly neutrophils.

Temporal profiling of the APR demonstrated a significant disconnect between the hepatic expression of IL-1β and IL-1RA in response to SCI, with IL-1β peaking at 2 hours, but IL-1RA not until 6 hours post-injury. Liu et al. [27] observed a similar dissociation between IL-1β and IL-1RA expression in the injured spinal cord itself, with IL-1β being elevated at 6 hours post-SCI, whilst IL-1RA protein was decreased; a significant increase in IL-1RA protein was observed at 24 hours post-SCI. This delay in IL-1RA expression suggests the existence of putative therapeutic window during which suppression of excessive IL-1β signalling in both the liver and/or spinal cord might be beneficial. Indeed, IL-1β is known to plays a detrimental role in the progression of CNS injury [18], and IL-1 knock out (KO) mice show improved locomotor activity, reduced lesion volumes and cell survival after SCI [20, 44]. Further data by Boato and colleagues [45] demonstrated that direct application of IL-1β to the spinal cord lesion site worsened neurological outcome. Moreover, injection of IL-1β into the spinal cord induced neutrophil recruitment [6]. Acute targeting of IL-1 signalling is thus likely to be a viable strategy to reduce secondary damage after SCI [22, 26].

IL-1RA treatment to dampen IL-1 signalling has previously proven neuroprotective in animal models of both stroke [31, 42, 46, 47] and TBI [28–30], leading to its progression into human clinical trials for these acquired CNS injuries [48–50]. In comparison to brain injuries, treatment of SCI with IL-1RA has been significantly under investigated. Several studies have applied IL-1RA locally to the lesion site itself and reported histological and motor function improvements [27, 32, 51, 52], however, none investigated the influence of this intervention on the APR. Similarly, despite administrating IL-1RA systemically, the studies by Li et al. [33] and Hasturk et al. [34] both focused on neurological endpoint markers such as spinal cord lesion size, cell death and/or apoptosis. Our study is therefore the first to explore the role of IL-1 signalling in the hepatic APR and how peripheral administration of IL-1RA affects both the systemic and central inflammatory response to SCI. Perhaps somewhat counterintuitively at first sight, IL-1β expression in the liver was increased after IL-1RA of SCI mice, but this is likely a compensatory response to its blockade. In another acute CNS injury study, it has been shown that the peripheral neutralisation of TNF after stroke gives rise to an increase in hepatic TNF expression, yet the number of granulocytes in the infarct was reduced [53]. Although the underlying mechanisms driving the increase in IL-1β expression following IL-1RA administration are unknown, the critical aspect is that the downstream IL-1-mediated signalling itself is suppressed. Hepatic CXCL1 and SAA-2 expression, which are induced by IL-1β [5], were indeed decreased, as was neutrophil infiltration into the livers of SCI mice with IL-1RA treatment, which has also been shown to be IL-1β-dependent [54]. Inhibition of IL-1β with IL-1RA during the hyperacute phase after injury thus significantly abrogates the overall hepatic APR.

Exogenous IL-1RA also significantly reduced early neutrophil infiltration to the injured spinal cord. Given the known links between the magnitude of the APR, associated leukocyte mobilisation, and outcome from CNS insults [3, 9, 55], it is tempting to speculate that this reduction in neutrophil recruitment with systemic IL-1RA treatment is due, at least in part, to the observed suppression of the APR. Targeting peripheral inflammation has indeed been demonstrated to be a promising strategy in other CNS injuries, where depletion of liver Kupffer cells [56] and the targeting of acute phase proteins [10] reduced both neutrophil infiltration and lesion size. Conversely, systemic IL-1β administration was shown to worsen neutrophilia and increase infiltration of these cells into the brain in a rodent model of stroke [54]. Interestingly, systemic IL-1β administration no longer had a detrimental effect on lesion pathology if neutrophils were depleted [54]. This demonstrates that IL-1β-mediated neutrophil activation and recruitment is the driving factor for excessive CNS damage after injury, and supports the notion that peripheral effects of IL-1RA convey neuroprotection via suppression of this recruitment. These findings have translational significance as the magnitude of neutrophilia is predictive of outcomes in both mice and human patients [36], and attenuating neutrophil recruitment to the injured spinal cord leads to improved recovery [57]. Targeting the APR thus provides an appealing strategy to reduce the systemic inflammatory response to SCI and, in doing so, ameliorate the spread of secondary injury into spared neural tissue around the impact site. Whilst only female mice have been used in this study, owing to the reduced risk of post-surgery complications, the population of those living with SCI is very heterogeneous. Further studies are therefore warranted to include animals of both sexes and of different ages, to determine whether these factors might affect the response to the IL-1RA treatment.

It must be acknowledged that exogenous IL-1RA may have had some direct effect on the spinal cord itself. Previous studies investigating the pharmacokinetics of IL-1RA report its ability to cross the blood-CNS barrier [58, 59], although uptake of IL-1RA by the CNS is normally extremely poor at around only 1-2% of the plasma concentration [59]. That said, breakdown of the blood-spinal cord barrier after SCI may allow for its passive accumulation here. Pradillo et al. [31] showed that delayed IL-1RA treatment after stroke reduced infarct volume and also neutrophil infiltration, which was attributed to IL-1RA inhibiting cytokine expression in microglia. The reduction in neutrophil accumulation in the injured spinal cord, as observed in this study, could therefore also be due to direct local suppression of IL-1β signalling at the lesion site. However, the temporal kinetics of the APR and neutrophil mobilisation response to SCI, which precedes the recruitment of these cells to the lesion site and also the peak of IL-1β expression here [22], strongly suggest that a significant aspect of the therapeutic efficacy of IL-1RA treatment that we observed here was mediated through targeting the peripheral inflammatory response.

## Conclusion

In summary, we show that peripheral administration of IL-1RA following SCI attenuates the APR and reduces neutrophil recruitment to the lesion site. Whether this effect is exerted directly or indirectly by suppression of the APR remains the focus of our ongoing research. Considering that IL-1RA treatment is already in clinical trials for other types of acquired CNS injury, our results warrant longer term studies to further explore its use as a viable and translatable treatment for traumatic SCI patients.

## Abbreviations

ANOVA: Analysis of variance
APR: Acute phase response
APPs: Acute phase proteins
BSA: Bovine serum albumin
CNS: Central nervous system
DAB: 3,3’-Diaminobenzidine
IL-1β: Interleukin-1β
IL-1RA: IL-1 receptor antagonist
kdyne: Kilodyne
KO: Knock out
OCT: Optimal cutting temperature compound
RBC: Red blood cell
RT: Room temperature
(D)PBS: (Dulbecco’s) phosphate buffered saline
SCI: Spinal cord injury
SEM: Standard error of the mean
SIRS: Systemic inflammatory response syndrome
TBI: Traumatic brain injury
